# Evaluating Subcriticality during the Ebola Epidemic in West Africa

**DOI:** 10.1371/journal.pone.0140651

**Published:** 2015-10-20

**Authors:** Wayne T. A. Enanoria, Lee Worden, Fengchen Liu, Daozhou Gao, Sarah Ackley, James Scott, Michael Deiner, Ernest Mwebaze, Wui Ip, Thomas M. Lietman, Travis C. Porco

**Affiliations:** 1 Department of Epidemiology and Biostatistics, University of California San Francisco, San Francisco, California, United States of America; 2 Francis I. Proctor Foundation for Research in Ophthalmology, University of California San Francisco, San Francisco, California, United States of America; 3 Mathematics and Statistics, Colby College, Waterville, Maine, United States of America; 4 Department of Ophthalmology, University of California San Francisco, San Francisco, California, United States of America; 5 Makerere University, Kampala, Uganda; The Australian National University, AUSTRALIA

## Abstract

The 2014–2015 Ebola outbreak is the largest and most widespread to date. In order to estimate ongoing transmission in the affected countries, we estimated the weekly average number of secondary cases caused by one individual infected with Ebola throughout the infectious period for each affected West African country using a stochastic hidden Markov model fitted to case data from the World Health Organization. If the average number of infections caused by one Ebola infection is less than 1.0, the epidemic is subcritical and cannot sustain itself. The epidemics in Liberia and Sierra Leone have approached subcriticality at some point during the epidemic; the epidemic in Guinea is ongoing with no evidence that it is subcritical. Response efforts to control the epidemic should continue in order to eliminate Ebola cases in West Africa.

## Introduction

The 2014–2015 Ebola outbreak in West Africa is already the largest on record. Beginning with a case in Guinea in December 2013 [1], the outbreak extended to Liberia, Sierra Leone, Nigeria, and Senegal [2]. Activities, such as early diagnosis, patient isolation and care, contact tracing, infection control practices, and safe burials, are recommended strategies to reduce transmission and control the epidemic [3] and have been implemented in the affected countries. Although there have been reports citing evidence for a decrease in transmission of Ebola virus after the implementation of prevention and response measures [4–6], the precise time when control of an epidemic has been achieved is difficult to determine during the course of the epidemic. A degree of control has been achieved when, on average, one infectious case does not produce another infectious case. That is, the disease has become subcritical and can no longer sustain itself in the population [7].

We examined international reported Ebola case data to determine if and when the Ebola epidemic became subcritical in Liberia, Sierra Leone, or Guinea. Using a stochastic hidden Markov model to simulate the epidemic through time, we estimated the weekly average number of infections caused by one Ebola infection within each of the three affected countries in West Africa using country and sub-country confirmed case counts available from the World Health Organization (WHO).

## Methods

### Data Sources

Weekly country- and subcountry-specific confirmed case count data were obtained from the WHO Global Health Observatory's patient database [8]. The number of confirmed cases using standard surveillance definitions [9] were given by week for Liberia, Sierra Leone, and Guinea from January 5, 2014 to July 29, 2015, if available. We used the data released by the World Health Organization on July 29, 2015 for the final analysis.

### Model Details

The basic reproduction number, *R*_*0*_, the average number of secondary cases caused by one infected individual throughout the infectious period in a completely susceptible population, has been used to monitor epidemics of infectious diseases [10] including Ebola, with particular interest in when it decreases below 1.0 [11]. Time-varying estimates of the reproduction number *R* for the epidemics in Liberia, Sierra Leone, and Guinea were calculated using a stochastic hidden Markov model. To assess the ongoing weekly transmission potential of Ebola in the three most intensely affected countries, we analyzed a simple mathematical transmission model based on previously published simple models [11]. The model consists of SEIR-type state spaces with two sequential exposure classes with progression rates chosen to provide a gamma-distributed incubation period of 10 days with a shape parameter of 2 (to be consistent with a previous WHO model [2]). The model distinguishes diagnosed from undiagnosed (and unknown) infectious cases with undiagnosed cases becoming diagnosed at a given rate. The transmission rates for undiagnosed and diagnosed cases are assumed to be different with diagnosed cases having a lower transmission rate due to case recognition and implementation of control measures. The number of confirmed cases is a counting process and is represented by a component of the state vector. Specifically, the states of the model are (1) uninfected individuals, (2, 3) latent or exposed individuals (infected, but who have not shown symptoms yet), (4) undiagnosed cases, (5) diagnosed cases, (6) removed individuals (died or recovered), and (7) confirmed cases. A full specification of the model is given in the S1 Appendix.

The number of susceptible individuals, exposed individuals of different classes, undiagnosed cases, diagnosed cases, and removed individuals together determine the counting process for the number of confirmed cases, which is all that is assumed to be observed. The model is simulated as a continuous-time Markov chain. Based on the state of the system at the beginning of any given week (*X* ^(t)^), the model is projected forward with the SEIR model, yielding the state at the end of the week *X* ^(t+1)^. The number of new confirmed cases predicted by the model is computed for that week (see S1 Appendix for details). This procedure is repeated *n* times, yielding a simulation-based estimate of the number ${\hat{C}}_{t}$ of new cases predicted, given the state and parameter values. We use a simple nonlinear particle filtering method to fit the simulated results to the WHO data (similar to the basic particle filter but using the resample/move method to prevent particle diversity depletion [12]). A Monte Carlo likelihood of the observed data given parameter values is calculated according to a Poisson distribution with ${\hat{C}}_{t}$ as the rate parameter. An alternate likelihood was derived by directly estimating the probability that the simulated number of confirmed cases exactly matched the observed number (the relative frequency of exact matches in the number of replications, listed as Bernoulli in the table in the S1 Appendix). This procedure, in turn, is repeated for each of *P* particles, consisting of state and parameter values. Each particle yields a Monte Carlo likelihood *L*_*j*,*t*_ of the observed data given a set of parameters at time *t*. Importance resampling is then used, wherein the particles are resampled with weight $w_{j,t}=L_{j,t}/\sum_{j\prime }L_{j\prime ,t}$ [13, 14] to choose the parameter values that most likely fit the data. To prevent depletion of particle diversity over time due to successive resampling, the resample/move method [15] is used. After resampling, the parameter values for each particle are perturbed using a symmetric transition kernel. Then, the original values of *X* ^(t)^ for that particle are used and the projection recomputed with new particles, leading to a new value for the Monte Carlo likelihood, *L*'. The perturbed values are accepted with probability *r* = min(1,*L*′/*L*) (i.e., a single step of the Metropolis algorithm). The procedure is iteratively repeated at each time point. Once a fitted set of parameters are obtained, an estimated *R* can be calculated (see below).

Previous authors have observed the non-identifiability of specific parameter sets for the Ebola process [16, 17], and our model is not identifiable either. We assign values for all parameters except for the transmission coefficient *β*, and conduct the fitting algorithm (see Shaman [18]). Of particular importance is the fraction of unconfirmed cases since an arbitrarily changing pattern of incomplete reporting is indistinguishable from changes in transmission. Note that if transmission increases or decreases over time, the resample/move procedure introduces perturbations in the parameters and provides the ability to track changes dynamically. Given the parameter choices, we then compute the instantaneous reproduction number (*R*) implied by the model, which for our simple model, is given by

$$R_{}=\beta \left(\frac{1}{\mu +\rho +\sigma }+\frac{\kappa }{\mu +\rho }\right)$$

where *κ* is the relative infectivity of diagnosed cases, *μ* is the mortality rate, *ρ* is the recovery rate, σ is the diagnosis rate, and *β* is a transmission coefficient. More generally, we should estimate the effective reproductive number by multiplying this quantity by the fraction of the population who are susceptible. However, by assumption, in this model, all immune individuals are former cases, and yet the cumulative incidence is small compared to the total population of each country, and we assume the susceptible fraction is essentially 1. Thus, assessment of control using this estimated *R* is somewhat pessimistic, since it is quite possible for the effective reproduction number to be less than one while our estimated *R* >1.

### Ethics Statement

For this study, we used weekly country- and subcountry-specific confirmed case count data that were obtained from the WHO Global Health Observatory's patient database available on the Internet [8]. Since the research only involved unidentifiable summary data (i.e., case counts by week), the research did not require review by the Committee on Human Research at the University of California, San Francisco.

## Results

**Fig 1:**
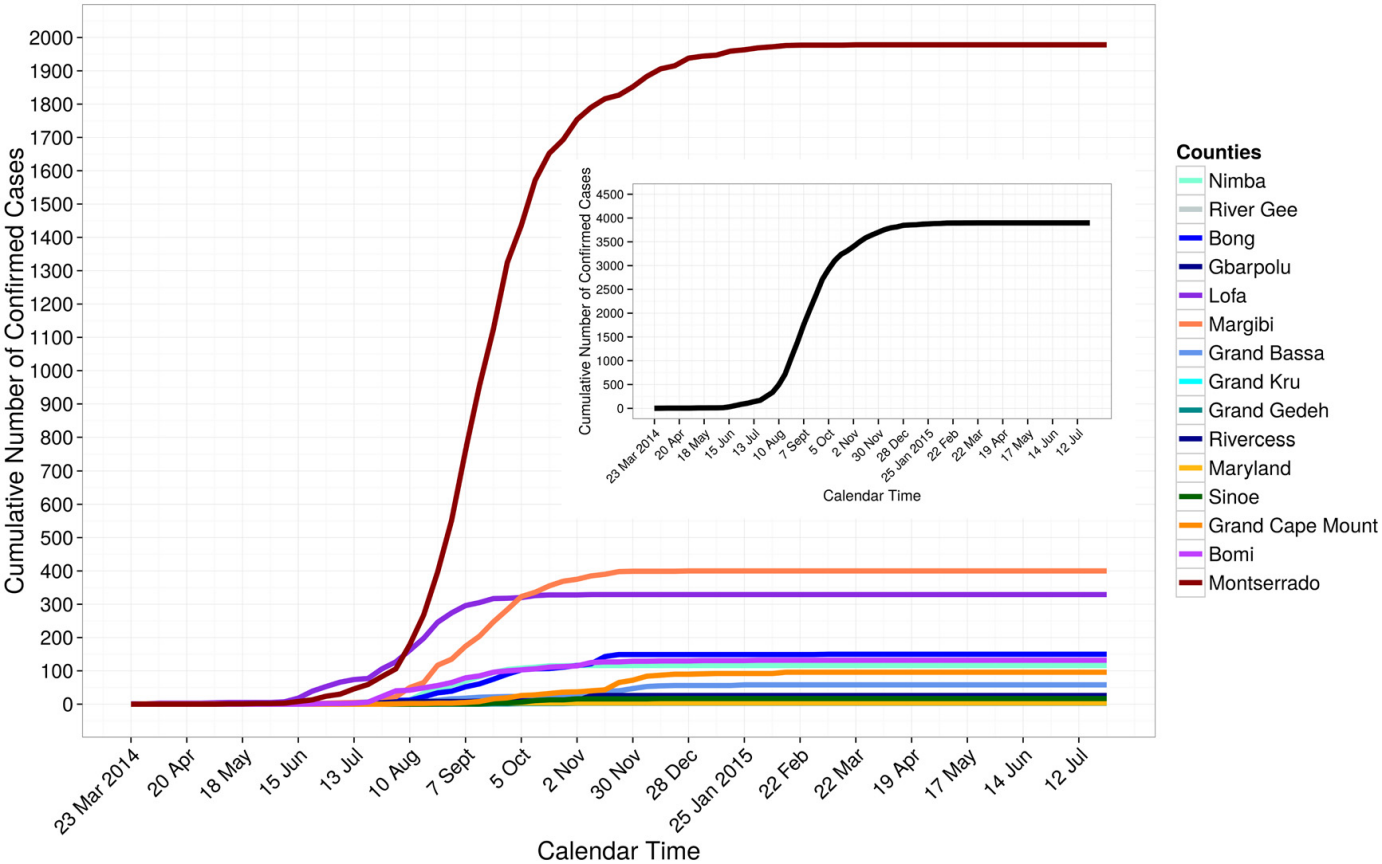
Cumulative number of confirmed cases in Liberia, March 23, 2014 to July 26, 2015. The cumulative numbers of confirmed cases over time for each of the 15 counties are shown using different colors. The cumulative number of confirmed cases over time for all 15 counties of the country is shown by the black line in the figure inset.

**Fig 2:**
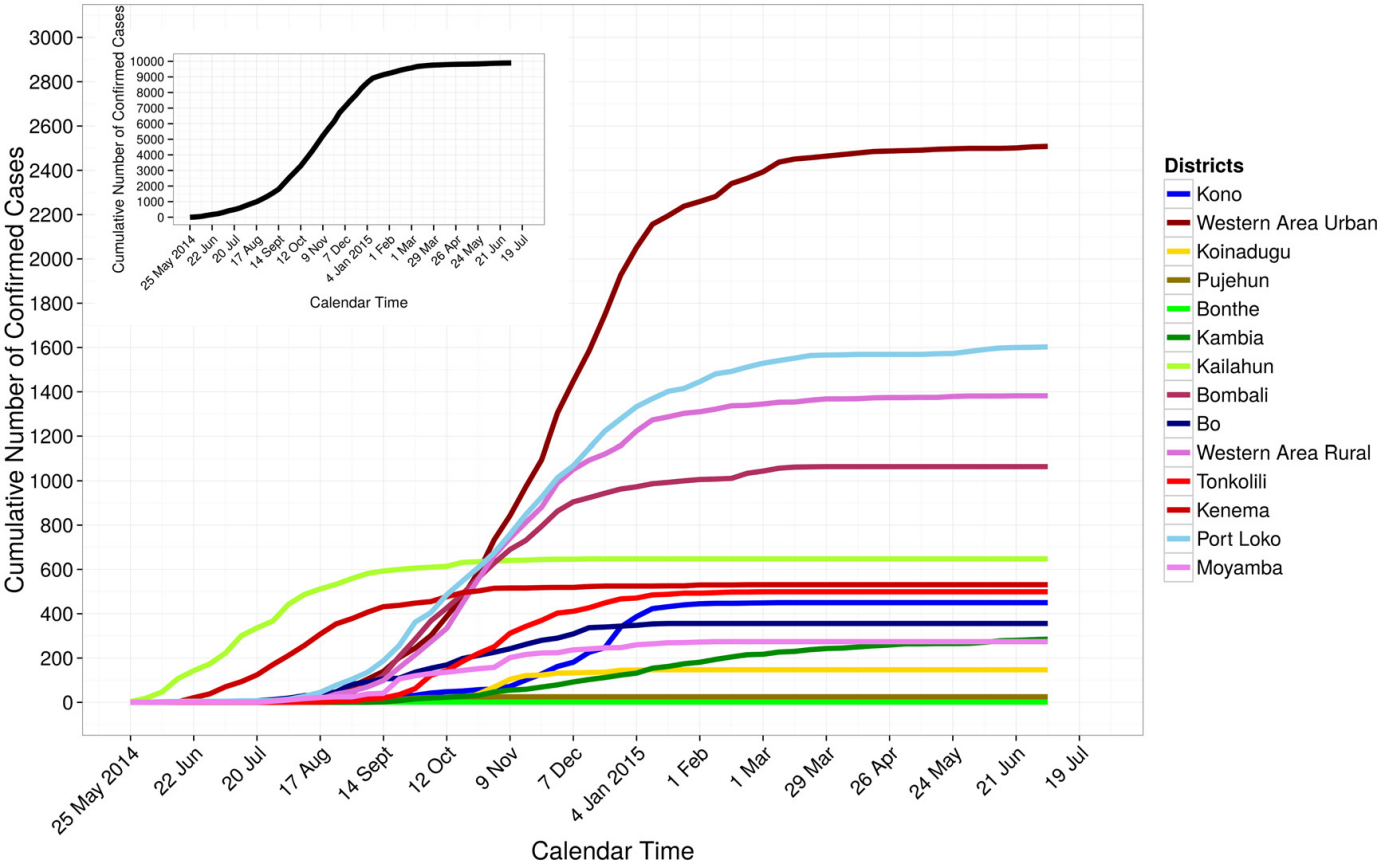
Cumulative number of confirmed cases in Sierra Leone, May 25, 2014 to July 5, 2015. The cumulative numbers of cases over time for each of the 14 districts are shown using different colors. The black line in the figure inset shows the cumulative number of cases over time for all 14 districts of the country.

**Fig 3:**
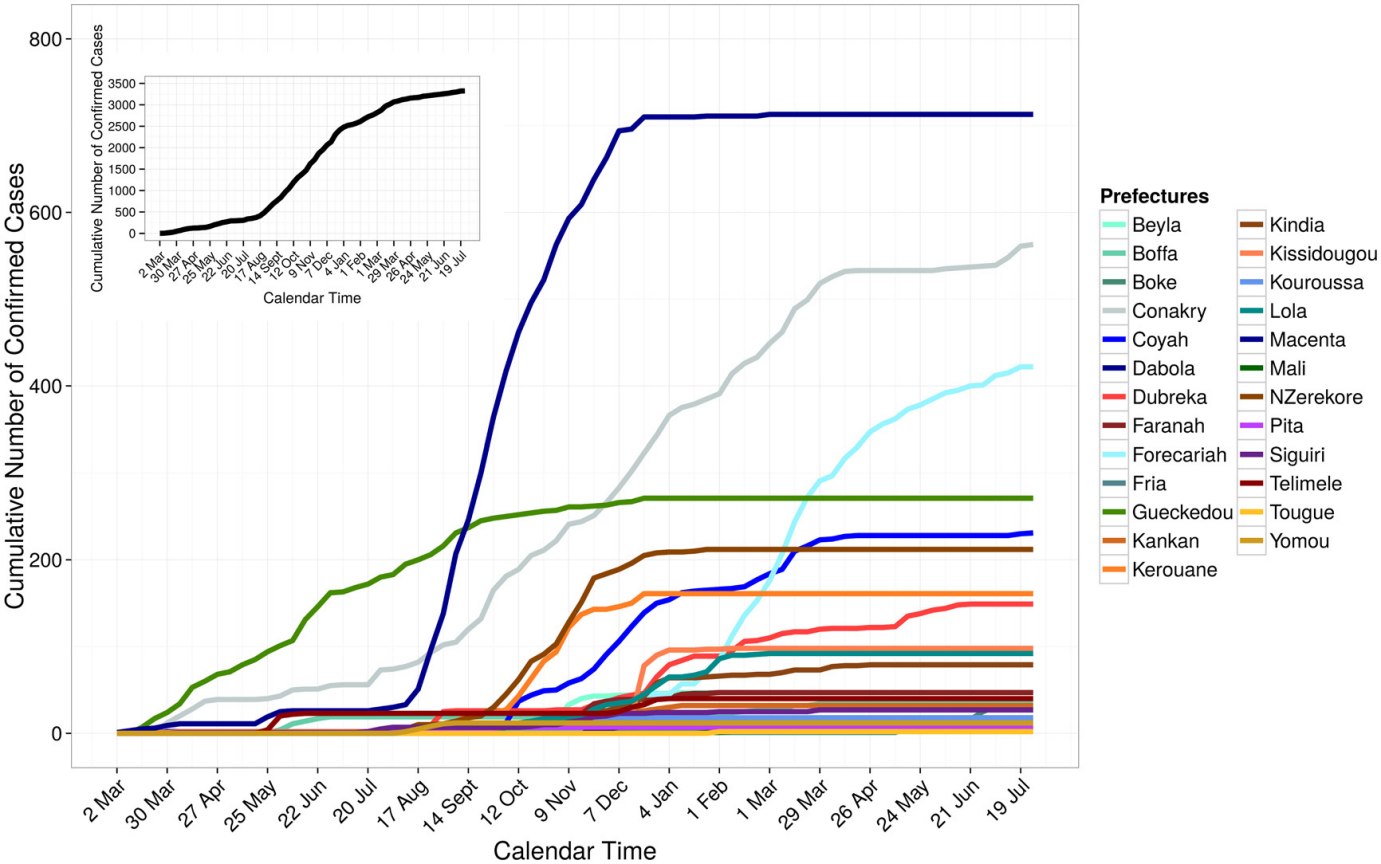
Cumulative number of confirmed cases in Guinea, March 2, 2014 to July 26, 2015. The cumulative number of cases over time for each of the 25 prefectures that reported data are shown using different colors. The black line in the figure inset shows the cumulative number of cases over time for 25 prefectures in the country.

There were 3,897 Ebola confirmed cases in Liberia as of July 26, 2015 (Fig 1). The weekly number of incident confirmed cases ranged from 0 to 364; 364 incident cases were reported during the week of September 1 to September 7, 2014. Montserrado County had the largest number of cumulative confirmed cases in the country (1,978 cases). In Sierra Leone, there were 9,896 confirmed cases as of July 5, 2015 (Fig 2). The weekly number of incident confirmed cases ranged from 3 to 578 with the largest number reported during the week of November 24 to November 30, 2014. The capital city of Freetown (Western Area Urban District) had the largest number of cumulative confirmed cases (2,508 cases). Guinea had 3,325 confirmed cases as of July 26, 2015 (Fig 3). The weekly number of incident confirmed cases ranged from 1 to 171, with the largest number of reports occurred during the week of December 15 to December 21, 2014. The Macenta prefecture had the highest number of reported cumulative confirmed cases (713 cases).

**Fig 4:**
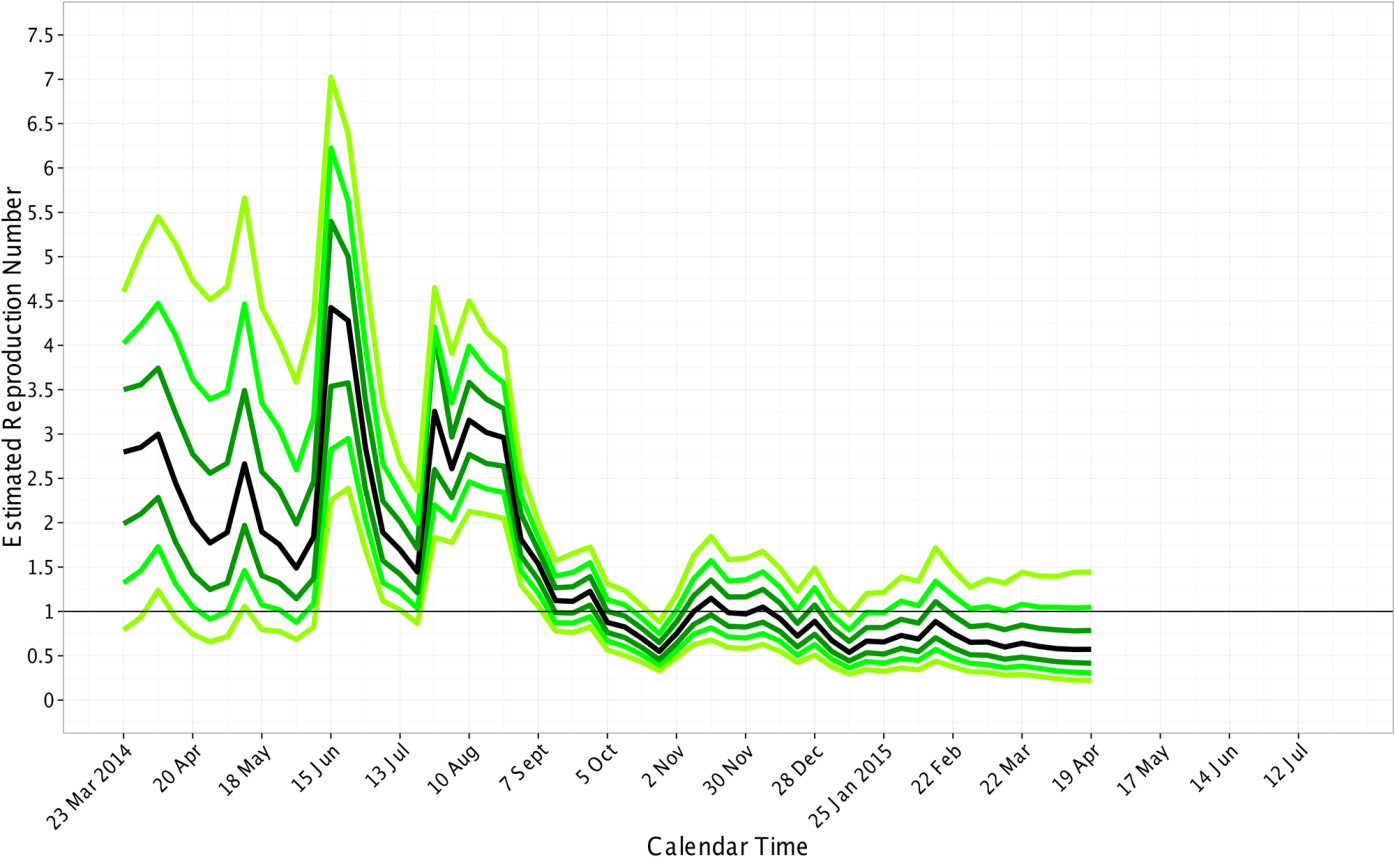
Estimated Reproduction Number over calendar time for Liberia. The quantiles of the reproduction number are displayed on the last day for each week. From bottom to top, the quantiles are 0.025, 0.10, 0.25, 0.50 (black line), 0.75, 0.90, and 0.975. The black horizontal line shows the threshold for subcriticality, i.e., the reproduction number equal to 1.0. We analyzed data through June, 2015; we did not include 6 cases that were reported in the situation reports during the weeks ending on July 5, 2015 and July 12, 2015.

**Fig 5:**
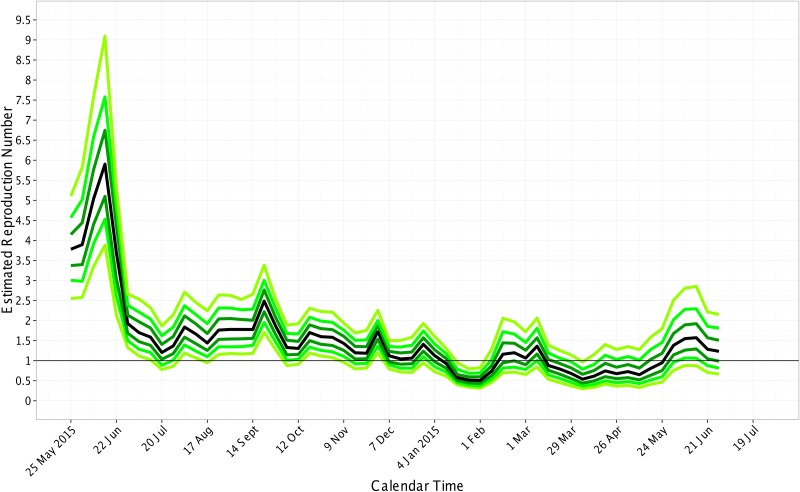
Estimated Reproduction Number over calendar time for Sierra Leone. The quantiles of the reproduction number are displayed on the last day of each week. From bottom to top, the quantiles are 0.025, 0.10, 0.25, 0.50 (black line), 0.75, 0.90, and 0.975. The black horizontal line shows the threshold for subcriticality, i.e., the reproduction number equal to 1.0.

**Fig 6:**
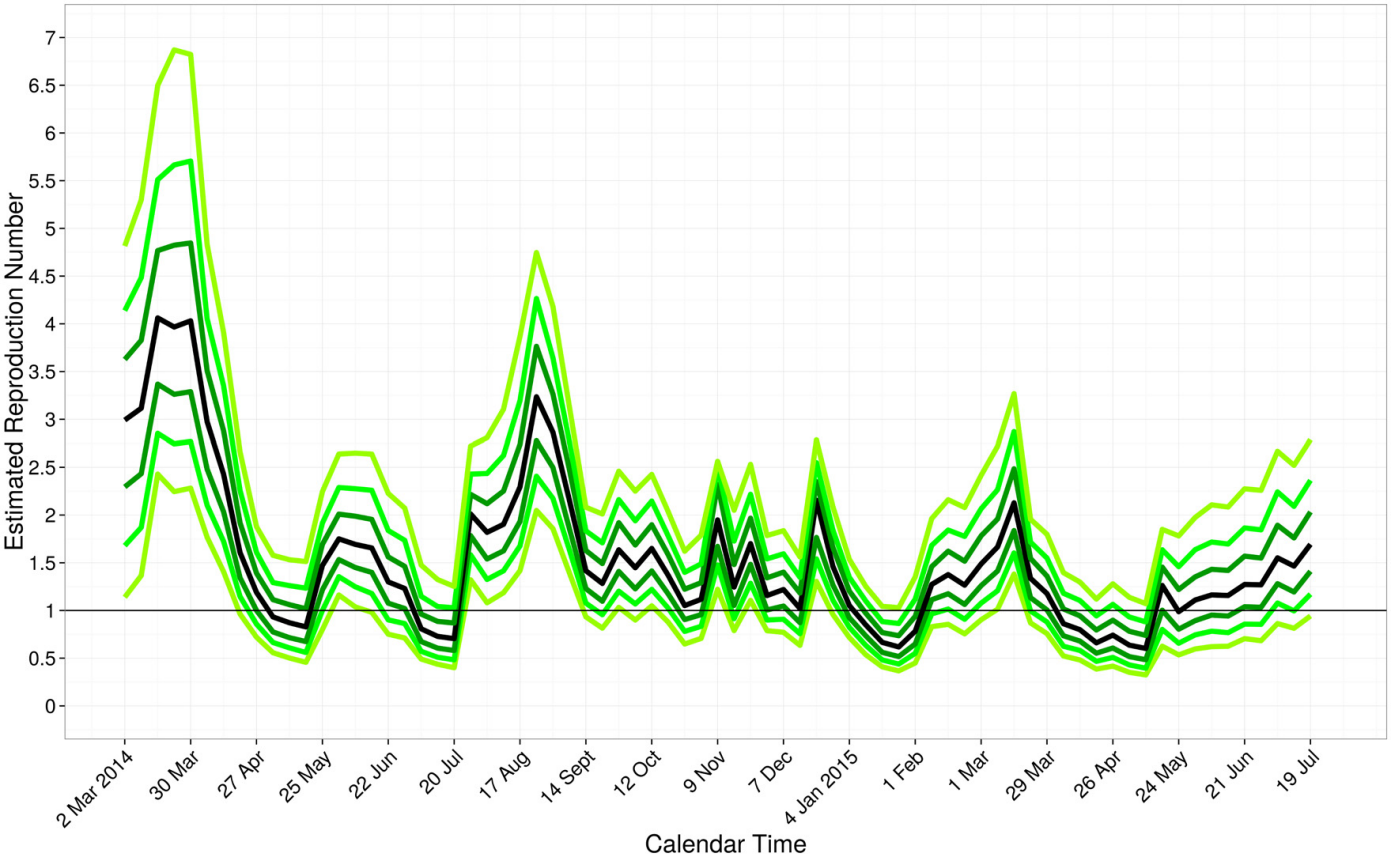
Estimated Reproduction Number over calendar time for Guinea. The quantiles of the reproduction number are displayed on the last day of each week. From bottom to top, the quantiles are 0.025, 0.10, 0.25, 0.50 (black line), 0.75, 0.90, and 0.975. The black horizontal line shows the threshold for subcriticality, i.e., the reproduction number equal to 1.0.

To be conservative, we considered an epidemic to be subcritical when the upper bound of the 95% credible interval for *R* dropped below 1.0. In Liberia, the median *R* ranged from 0.9 to 4.6 between March 23, 2014 and October 5, 2014 (Fig 4). The estimate of *R* was subcritical on October 26, 2014; although the median *R* estimates after November 30, 2014 are below 1.0, the upper bound is greater than 1.0. In Sierra Leone, the median *R* ranged from 0.6 to 5.9 between May 25, 2014 and January 18, 2015 (Fig 5). The time-varying estimates of *R* reached subcriticality in Sierra Leone during the week ending on January 18, 2015 to February 1, 2015. These estimates are consistent with a previous report that the epidemic in Sierra Leone was subcritical as of January 18, 2015 [19]. In Guinea, the epidemic was not subcritical from March 2, 2014 through July 19, 2015 (Fig 6).

## Discussion

This study suggests that the epidemics in Liberia and Sierra Leone may have reached subcriticality in October 2014 and late January and early February 2015 respectively. As the number of cases declined and the concerns of its implications for vaccine studies grew [20], our results showed that the latest median estimate of *R* for Liberia was less than 1.0 with the last confirmed case reported during the week ending March 22, 2015 in the patient database. Similarly, the estimates of *R* for Sierra Leone may be declining as of late June 2015. Interventions and response efforts to control the disease must continue to make the epidemic subcritical in the two affected countries with reported cases, despite previous observations that transmission may be decreasing. However, we recognize that epidemic outcomes depend on individual behaviors as well as hospital capacities to care for sick, infectious, and dying individuals [21]; a multi-faceted control approach is most certainly warranted [22].

Other researchers have published estimated transmission characteristics at various time points in the epidemic. A three-month ensemble forecast produced estimates of the basic reproduction number between 1.5 and 2.0 using data from July 6, 2014 to August 9, 2014 and a stochastic, individual-based model that provided estimates for the local transmission of Ebola virus [23]. Using a set of ordinary differential equations, basic reproduction numbers were estimated to be 1.6 for Liberia, 2.5 for Sierra Leone, and 1.5 for Guinea using data as of August 20, 2014 [24], which are consistent with other *R*_*0*_ estimates between 1.6 and 2.0 for the same time period [25]. With control measures, the effective reproductive number dropped to around 1.0 in Guinea and Sierra Leone by the end of May and July 2014, respectively using different methods. In Liberia, the effective reproductive number remained unchanged suggesting that there was no evidence of outbreak control during the same period of time [24]. Subsequently, the WHO Ebola Response Team estimated the basic reproduction numbers as 1.8 for Liberia, 2.0 for Sierra Leone, and 1.7 for Guinea using data as of September 14, 2014 [2]. Using data from July to September, 2014, the average number of secondary infections per infected individual was estimated to be 1.73, with survivors having a different estimate in comparison with non-survivors (0.66 and 2.36 respectively) in Montserrado County, Liberia [26]. Additional effective *R* estimates for the same time period ranged from 1.3 to 2.7 among the three west African countries [27].

By mid-October 2014, there were suggestions of a change in the growth of the epidemic in Liberia [17]; one model showed that the exponential growth phase that was observed in Liberia previously was over, an observation that was consistent with epidemiologic data known at that time [17]. Using different methods, our results suggest that *R* became subcritical in Liberia in late October 2014. In Sierra Leone, *R* became subcritical in late January to early February 2015, confirming a previous *R* estimate [19]. Driving *R* to be less than 1.0 would certainly control the epidemic, but epidemics often fade out stochastically even when *R* is approximately 1.0 [28]. In order to control the epidemic, a combined approach involving different control strategies (i.e., case isolation, contact-tracing with quarantine, and safe funeral and burial practices) must be implemented [22]. It is not possible, however, to disentangle changes in reporting from changes in transmission. We also note that the wide credible limits on the reproduction number seen near the end of the time series may reflect, in part, low power resulting from small case counts.

An *R* estimate below 1.0 for any of the three epidemics would suggest the possibility of elimination, but certainly would not imply that no further cases will occur [29]. In addition, our analyses incorporated data that were available on July 29, 2015. If the last few weeks of data change retrospectively due to reporting delays, i.e., more cases are added to the last few weeks of the closing date of the data, our estimates of *R* during this time period will change. An important limitation of the simple model we have chosen is that geographic heterogeneity has not been explicitly represented; ongoing transmission in specific regions or “hot spots” could be occurring even though the basic reproduction number estimated from a simple homogeneous model is less than 1.0. Further work is therefore needed to include the effects of geographic heterogeneity as has been published previously [30, 31], including an assessment of the time to complete elimination. Also, our model also does not account for clustered transmission as has been noted to occur [32] or account for the effects of ring vaccination [33].

## Conclusions

In summary, subcriticality of an epidemic is an important indicator that a degree of control has been achieved before the absence of Ebola cases. The epidemics in Liberia and Sierra Leone have approached subcriticality at some point during the epidemic; the epidemic in Guinea is ongoing with no evidence that it is subcritical. In our view, this suggests that the national and international responses must continue if true elimination is to occur throughout West Africa.

## Supporting Information

S1 Appendix Details of the Model. (DOCX)

## References

[1] Ebola: a failure of international collective action. Lancet. 2014; 384: 637 25150744 10.1016/S0140-6736(14)61377-5

[2] WHO Ebola Response Team. Ebola Virus Disease in West Africa—The First 9 Months of the Epidemic and Forward Projections. N Engl J Med. 2014;.371(16): 1481–95. 10.1056/NEJMoa1411100 25244186 PMC4235004

[3] World Health Organization. “Ebola Response Roadmap,” (available at http://apps.who.int/iris/bitstream/10665/131596/1/EbolaResponseRoadmap.pdf?ua=1).

[4] Sharma A, Heijenberg N, Bolongei J, Reeder B, Alpha T, Sterk E, et al. Evidence for a decrease in transmission of Ebola virus—Lofa County, Liberia, June 8-November 1, 2014. Morb Mortal Wkly Rep. 2014;63: 1067–1071. PMC5779501 25412065

[5] Nyenswah TG, Westercamp M, Kamali AA, Quin J, Zielinski-Gutierrez E, Amegashie F, et al. Evidence for declining numbers of Ebola cases—Montserrado County, Liberia, June-October 2014. Morb Mortal Wkly Rep. 2014; 63:1072–1076. PMC5779509 25412066

[6] Nyenswah T, Fahnbulleh M, Massaquoi M, Nagbe T, Bawo L, Falla JD, et al Ebola epidemic—Liberia, March-October 2014. Morb Mortal Wkly Rep. 2014;63: 1082–1086. PMC5779504 25412068

[7] Halloran ME, Concepts of transmission and dynamics In: Thomas JC, Weber DJ, editors. Epidemiological Methods for the Study of Infectious Diseases. Oxford: Oxford Press; 2001.

[8] World Health Organization Global Health Observatory. Ebola data and statistics. 2014. Available at http://apps.who.int/gho/data/node.ebola-sitrep.ebola-country?lang=en.

[9] World Health Organization. Case definition recommendations for Ebola or Marburg virus diseases. 2014. Available at http://www.who.int/csr/resources/publications/ebola/ebola-case-definition-contact-en.pdf?ua=1.

[10] Wallinga J, Teunis P. Different epidemic curves for severe acute respiratory syndrome reveal similar impacts of control measures. Am J Epidemiol. 2004;160: 509–516. 15353409 10.1093/aje/kwh255 PMC7110200

[11] Chowell G, Nishiura H, Transmission dynamics and control of Ebola virus disease (EVD): a review. BMC Med. 2014;12: 196 10.1186/s12916-014-0196-0 25300956 PMC4207625

[12] Yang W, Karspeck A, Shaman J. Comparison of filtering methods for the modeling and retrospective forecasting of influenza epidemics. PLoS Comput Biol. 2014;10: e1003583 10.1371/journal.pcbi.1003583 24762780 PMC3998879

[13] Gordon NJ, Salmond DJ, Smith AFM. Novel approach to nonlinear/non-Gaussian Bayesian state elimination. IEEE Proceedings-F. 1993;140: 107–113.

[14] Kitagawa G. A self-organizing state-space model. J Am Stat Assoc. 1998;93: 1203–1215.

[15] Gilks WR, Berzuini C. Following a moving target—Monte Carlo inference for dynamic Bayesian models. J R Stat Soc Ser B (Statistical Methodol). 2001;63: 127–146.

[16] Weitz JS, Dushoff J. Modeling post-death transmission of Ebola: challenges for inference and opportunities for control. Sci Rep. 2015;5: 8751 10.1038/srep08751 25736239 PMC4348651

[17] Chowell G, Simonsen L, Viboud C, Kuang Y. Is west Africa approaching a catastrophic phase or is the 2014 Ebola epidemic slowing down? Different models yield different answers. PLoS Curr. 2014 11 20;6 10.1371/currents.outbreaks.b4690859d91684da963dc40e00f3da81 PMC4318911 25685615

[18] Shaman J, Yang W, Kandula S. Inference and forecast of the current west African Ebola outbreak in Guinea, Sierra Leone and Liberia. PLoS Curr. 2014 10 31;6 10.1371/currents.outbreaks.3408774290b1a0f2dd7cae877c8b8ff6 PMC4234409 25642378

[19] Camacho A, Kucharski A, Aki-Sawyerr Y, White MA, Flasche S, Baguellin M, et al Temporal changes in Ebola transmission in Sierra Leone and implications for control requirements: a real-time modelling study. PLoS Curr. 2015 2 10;7 10.1371/currents.outbreaks.406ae55e83ec0b5193e30856b9235ed2 PMC4339317 25737806

[20] Enserink M. The Ebola Epidemic. High hopes for Guinean vaccine trial. Science. 2015 1 16;347(6219): 219–20. 10.1126/science.347.6219.219 25593164

[21] Drake JM, Kaul RB, Alexander L, O'Regan SM, Kramer AM, Pulliam JT, et al Ebola cases and health system demand in Liberia. PLoS Biol. 2015 1 13;13(1): e1002056 10.1371/journal.pbio.1002056 25585384 PMC4293091

[22] Pandey A, Atkins KE, Medlock J, Wenzel N, Townsend JP, Childs JE, et al Strategies for containing Ebola in West Africa. Science. 2014;346: 991–5. 10.1126/science.1260612 25414312 PMC4316831

[23] Gomes MFC, Pastore y Piontti A, Rossi L, Chao D, Longini I, Halloran ME, et al Assessing the international spreading risk associated with the 2014 west African ebola outbreak. PLoS Curr. 2014 9 2;6 10.1371/currents.outbreaks.cd818f63d40e24aef769dda7df9e0da5 PMC4169359 25642360

[24] Althaus CL. Estimating the reproduction number of Ebola virus (EBOV) during the 2014 outbreak in west Africa. PLoS Curr. 2014 9 2;6 10.1371/currents.outbreaks.91afb5e0f279e7f29e7056095255b288 PMC4169395 25642364

[25] Fisman D, Khoo E, Tuite A. Early epidemic dynamics of the west african 2014 ebola outbreak: estimates derived with a simple two-parameter model. PLoS Curr. 2014 9 8;6 10.1371/currents.outbreaks.89c0d3783f36958d96ebbae97348d571 PMC4169344 25642358

[26] Yamin D, Gertler S, Ndeffo-Mbah ML, Skrip LA, Fallah M, Nyenswah TG, et al Effect of Ebola progression on transmission and control in Liberia. Ann Intern Med. 2015;162(1): 11–17. 10.7326/M14-2255 25347321 PMC4402942

[27] Towers S, Patterson-Lomba O, Castillo-Chavez C. Temporal variations in the effective reproduction number of the 2014 West Africa Ebola outbreak. PLoS Curr. 2014 9 18;6 10.1371/currents.outbreaks.9e4c4294ec8ce1adad283172b16bc908 PMC4169299 25642357

[28] Bailey NTJ. The elements of stochastic processes with applications to the natural sciences. New York: Wiley-Interscience; 1990.

[29] Blumberg S, Enanoria WTA, Lloyd-Smith JO, Lietman TM, Porco TC. Identifying postelimination trends for the introduction and transmissibility of measles in the United States. Am J Epidemiol. 2014 6 1;179(11): 1375–82. 10.1093/aje/kwu068 24786800 PMC4036219

[30] Merler A, Ajelli M, Fumanelli L, Gomes MF, Piontti AP, Rossi L, et al Spatio-temporal spread of the Ebola 2014 outbreak in Liberia and the effectiveness of non-pharmaceutical interventions: a computational modelling analysis. Lancet Infect Dis. 2015;15: 204–211. 10.1016/S1473-3099(14)71074-6. 25575618 PMC4409131

[31] Chowell G, Viboud C, Hyman JM, Simonsen L. The Western Africa ebola virus disease epidemic exhibits both global exponential and local polynomial growth rates. PLoS Curr. 2015 1 21;7 10.1371/currents.outbreaks.8b55f4bad99ac5c5db3663e916803261 PMC4322058 25685633

[32] Scarpino SV, Iamarino A, Wells C, Yamin D, Ndeffo-Mbah M, Wenzel NS, et al Epidemiological and viral genomic sequence analysis of the 2014 ebola outbreak reveals clustered transmission. Clin Infect Dis. 2015 4 1; 60(7): 1079–82. 10.1093/cid/ciu1131 25516185 PMC4375398

[33] Henao-Restrepo AM, Longini IM, Egger M, Dean NE, Edmunds WJ, Camacho A, et al Efficacy and effectiveness of an rVSV-vectored vaccine expressing Ebola surface glycoprotein: interim results from the Guinea ring vaccination cluster-randomised trial. Lancet. 2015 8 29;386(9996): 857–66. 10.1016/S0140-6736(15)61117-5 26248676

