# Correction: Evaluating Subcriticality during the Ebola Epidemic in West Africa

**DOI:** 10.1371/journal.pone.0157068

**Published:** 2016-06-02

**Authors:** Wayne T. A. Enanoria, Lee Worden, Fengchen Liu, Daozhou Gao, Sarah Ackley, James Scott, Michael Deiner, Ernest Mwebaze, Wui Ip, Thomas M. Lietman, Travis C. Porco

The following information is missing from the Funding section: TCP and EM gratefully acknowledge support from the Meaningful Modeling of Epidemiological Data (MMED) program, a NIH-funded joint initiative under the University of Florida, the South African Centre for Epidemiological Modelling and Analysis (SACEMA), and the African Institute for Mathematical Sciences (AIMS) (NIH NIGMS R25GM102149 to J.R.C. Pulliam and A. Welte).

## References

[1] Enanoria WTA, Worden L, Liu F, Gao D, Ackley S, Scott J, et al (2015) Evaluating Subcriticality during the Ebola Epidemic in West Africa. PLoS ONE 10(10): e0140651 doi: 10.1371/journal.pone.0140651 26484544 10.1371/journal.pone.0140651 PMC4618845

