# Supplementary material for: Evaluating Subcriticality during the Ebola Epidemic in West Africa
Source: PLoS One. 2015 Oct 20;10(10):e0140651. doi: 10.1371/journal.pone.0140651 (PMC4618845; doi:10.1371/journal.pone.0140651)
Supplement: S1 Appendix — (DOCX) [file pone.0140651.s001.docx]

**S1. Supporting Information: Appendix**

**Title:**

**Evaluating subcriticality during the Ebola epidemic in West Africa**

**Authors:**

Wayne T.A. Enanoria^1^, Lee Worden^2^, Fengchen Liu^2^, Daozhou Gao^2^, Sarah Ackley^2^, James Scott^2,3^, Michael Deiner^4^, Ernest Mwebaze^5^, Wui Ip^2^, Thomas M. Lietman^1,2,4^, Travis C. Porco^1,2,4*^

**Affiliations:**

^1^ Department of Epidemiology and Biostatistics, University of California at San Francisco, San Francisco, California, USA

^2^ Francis I. Proctor Foundation for Research in Ophthalmology, University of California at San Francisco, San Francisco, California, USA

^3^ Mathematics and Statistics, Colby College, Waterville, Maine, USA

^4^ Department of Ophthalmology, University of California at San Francisco, San Francisco, California, USA

^5^ Makerere University, Kampala, Uganda

* To whom correspondence should be addressed; Dr. Travis C. Porco, E-mail: travis.porco@ucsf.edu.

**Appendix**

***Data Sources***

Weekly country- and subcountry-specific confirmed case count data were obtained from the WHO Global Health Observatory's patient database [*8*] for Liberia, Sierra Leone, and Guinea from January 5, 2014 to July 29, 2015, if available. The analysis for Liberia included data through June 2015; the analysis did not include 6 confirmed cases that were reported in the situation reports during the weeks ending on July 5, 2015 and July 12, 2015.

***Model structure***

We examined a stochastic model of transmission. The state space is similar to previously published models in following susceptibles, latently infected individuals (two classes), infected individuals (diagnosed and undiagnosed), and removed individuals. For simplicity, we do not distinguish live from deceased infectives as has been done previously [*16*]. Only a fraction *f* of new diagnoses are reported as confirmed cases. The model obeys Markov transitions with intensities given in Table 1. The observations are modeled as a counting process such that each diagnosis (transition from $X_{4}$to $X_{5}$) is counted with probability *f*. Simulations were conducted with a time step of 1/16 day (which was varied in the sensitivity analysis). The number of confirmed diagnoses at time *t* is denoted $X_{7}^{(t)}$. The force of infection $\lambda(t)$ is given by $\lambda(t)=\beta\left(\frac{X_{4}}{N}+\kappa\frac{X_{5}}{N}\right)$, where *N* is the total number of individuals. Each diagnosed individual is counted as a confirmed case with probability *f*, the confirmation probability.

**Table 1. Model state space**

| Variable | State | Process | Transitions | Rate |
| --- | --- | --- | --- | --- |
| $X_{1}$ | Susceptible | Transmission | $X_{1}\to X_{2}$ | $\lambda(t)X_{1}$ |
| $X_{2}$ | Latent (1) | Progression | $X_{2}\to X_{3}$ | $\gamma(t)X_{2}$ |
| $X_{3}$ | Latent (2) | Progression | $X_{3}\to X_{4}$ | $\gamma(t)X_{3}$ |
| $X_{4}$ | Undiagnosed case | Diagnosis | $X_{4}\to X_{5}$ | $\sigma(t)X_{4}$ |
|  |  | Removal | $X_{4}\to X_{6}$ | $\mu(t)X_{4}$ |
| $X_{5}$ | Diagnosed case | Removal | $X_{5}\to X_{6}$ | $\mu(t)X_{5}$ |
| $X_{6}$ | Removed | -- |  |  |

A diagram of the model state spaces is given in Figure 1.

**Figure 1. Model state spaces**

**Sensitivity Analyses**

In addition, as a sensitivity analysis, we examined transmission at the regional level. In this case, we modeled the number of susceptibles, latent (stage 1), latent (stage 2), undiagnosed cases, diagnosed cases, and cumulative confirmed cases in each county or region. Here, we denote by $X_{I,j}$the number of susceptibles in region *j*, and so forth. The force of infection for region *j* becomes $\lambda_{j}\left(t\right)=\beta\left(\frac{X_{4,j}}{N_{j}}+k\frac{X_{5,j}}{N_{j}}\right)+b\beta\left(\frac{\sum_{j'\neq j} X_{4,j'}}{\sum_{j'\neq j} N_{j'}}+k\frac{\sum_{j'\neq j} X_{5,j'}}{\sum_{j'\neq j} N_{j'}}\right)$. The basic reproduction number is computed as the Perron-Frobenius eigenvalue of the next generation matrix [*27*].

Model parameters are listed in Table 2. The parameter $\gamma$ was defined to be *2(1/T)*, where *T* is the incubation period. The incubation period is modeled as a gamma distribution with a shape parameter of 2 (two exponential stages), approximating the single exposure incubation period reported by the WHO [*2*].

**Table 2. Model parameters**

| Parameter | Description | Base |
| --- | --- | --- |
|  |  | Value |
| $\beta$ | Transmission | Estimated |
| *T* | Incubation period | 10 days |
| $\sigma$ | Diagnosis rate | 0.3 day^-1^ |
| $\mu$ | Removal rate | 0.14 day^-1^ |
| $f$ | Reporting probability | 0.5 |
| $\kappa$ | Relative transmission for diagnosed cases | 1.0 |
| $b$ | Transmission multiplier between | -- |
|  | Regions |  |

The total removal rate is assumed to be 0.21 per day at baseline (derived from assuming a recovery rate of 0.07 per day (corresponding to approximately two weeks of illness), and a rate of death and burial of 0.14 per day (corresponding to approximately one week). However, it is understood that these parameters are poorly characterized, and so alternative scenarios were examined (see Table 3). Sensitivity analyses suggest the relative transmission for diagnosed cases and the reporting fraction of cases do not greatly affect the *R* estimate.

**Table 3. Sensitivity analysis for Liberia**

| Feature or Parameter | Change | Median estimate (95% credible interval) for final *R* estimate for Liberia (Week of March 2, 2015) |
| --- | --- | --- |
| Base case |  | 0.65 (0.32, 1.36) |
| Reporting fraction *f* | 0.5 to 0.25 | 0.63 (0.29, 1.34) |
| Reporting fraction *f* | 0.5 to 0.75 | 0.63 (0.231, 1.30) |
| Removal rate μ | 0.14 to 0.1 | 0.55 (0.24, 1.17) |
| Removal rate μ | 0.14 to 0.2 | 0.71 (0.34, 1.41) |
| Relative transmission k | 1.0 to 0.5 | 0.61 (0.29, 1.25) |
| Relative transmission k | 1.0 to 1.5 | 0.65 (0.29, 1.34) |
| Replications per particle | 128 to 512 | 0.65 (0.33, 1.23) |
| Data | Raw counts to three week centered moving average | 0.63 (0.28, 1.32) |
| Resolution | National to county level | 0.71 (0.35, 1.44) |
| Likelihood function | Poisson to Bernoulli | 0.63 (0.23, 1.32) |
| Change in diagnosis rate | Note 1 | 0.58 (0.25, 1.20) |
| Change in infectiousness for diagnosed individuals | Note 2 | 0.60 (0.27, 1.22) |
| Change in loss to follow-up | Note 3 | 0.65 (0.30, 1.40) |

Table 3 shows one example of a sensitivity analysis for Liberia, based on WHO data reported. In this example, we vary the reporting fraction from the base case value of 0.5 to 0.25 and to 0.75, and report the 95% credible interval for the basic reproduction number for Liberia. We similarly vary the removal rate and the relative transmission rate following diagnosis. We also conducted a sensitivity analysis replacing the data points by centered three week moving averages. Finally, the analysis was repeated using data at the county level, assuming a total between-county transmission of 1% that of within-county transmission. Note 1: Diagnosis rate assumed to increase from 0.3 to 0.5, linearly, beginning at day 150 (mid August 2014) and ending at day 200 (early October 2014). Note 2: Relative infectivity assumed to decrease from 1.0 to 0.5, linearly, beginning at day 200 (early October 2014) and ending at day 250. Note 3: Loss to follow up fraction assumed to decrease from 0.6 to 0.3, linearly, beginning at day 150 and ending at day 250 (late November 2014).

Each replication was conducted with approximately 2^12^ (4096) particles (except the county-level analysis was conducted with 2^13^=8192 particles, and runs with the binomial likelihood were conducted with 16384 particles). The model was implemented in Fortran 2008 (GNU Fortran 4.9), using Open MPI v. 1.8, and run on a Linux cluster with 64-core nodes.
